# pH Dependence of the Stress Regulator DksA

**DOI:** 10.1371/journal.pone.0120746

**Published:** 2015-03-23

**Authors:** Ran Furman, Eric M. Danhart, Monali NandyMazumdar, Chunhua Yuan, Mark P. Foster, Irina Artsimovitch

**Affiliations:** 1 Department of Microbiology, The Ohio State University, Columbus, Ohio, United States of America; 2 The Center for RNA Biology, The Ohio State University, Columbus, Ohio, United States of America; 3 Department of Chemistry and Biochemistry, The Ohio State University, Columbus, Ohio, United States of America; 4 Campus Chemical Instrument Center, The Ohio State University, Columbus, Ohio, United States of America; Baylor College of Medicine, UNITED STATES

## Abstract

DksA controls transcription of genes associated with diverse stress responses, such as amino acid and carbon starvation, oxidative stress, and iron starvation. DksA binds within the secondary channel of RNA polymerase, extending its long coiled-coil domain towards the active site. The cellular expression of DksA remains constant due to a negative feedback autoregulation, raising the question of whether DksA activity is directly modulated during stress. Here, we show that *Escherichia coli* DksA is essential for survival in acidic conditions and that, while its cellular levels do not change significantly, DksA activity and binding to RNA polymerase are increased at lower pH, with a concomitant decrease in its stability. NMR data reveal pH-dependent structural changes centered at the interface of the N and C-terminal regions of DksA. Consistently, we show that a partial deletion of the N-terminal region and substitutions of a histidine 39 residue at the domain interface abolish pH sensitivity in vitro. Together, these data suggest that DksA responds to changes in pH by shifting between alternate conformations, in which competing interactions between the N- and C-terminal regions modify the protein activity.

## Introduction

*Escherichia coli* DksA has been shown to play a key role in regulation of transcription of *r*ibosomal RNA and protein genes [1,2] and may also contribute to genome integrity by preventing conflicts between replication and transcription machineries [3]. *In addition*, *DksA*, *often in synergy* with the alarmone ppGpp, controls expression of a large number of genes required for motility [4,5], fimbriae biogenesis [6], pathogenesis [7,8], and stress responses to very diverse cellular signals, ranging from nutrient limitation [2] to oxidative and nitrosative damage [9]. Although ppGpp and DksA frequently function synergistically, examples of differential and even opposite regulation continue to accumulate [4,10,11]. Most strikingly, while both ppGpp and DksA are required for *rrnB* P1 regulation by many cellular signals [1,12], ppGpp is dispensable during phosphate starvation [10].

ppGpp and DksA bind to distant sites on the core RNA polymerase (RNAP) [13,14] and reduce the stability of the promoter complexes, leading to repression or activation of transcription depending on the properties of a target promoter [1,2]. Their most pronounced effect is to shut down synthesis of very abundant rRNAs by the σ^70^ holoenzyme, thereby potentially making core RNAP available for binding to alternative σ factors. This indirect control of σ factors activities is consistent with the core enzyme being the target of regulation and is supported by observations that reduced levels or affinity of σ^70^ for the core RNAP mimics the effect of ppGpp accumulation on activation of σ^S^ [15] and σ^54^ [16] transcription *in vivo*. However, recent data paint a more complex picture in which ppGpp and DksA regulate alternative σs directly [17] and differently [10]. While σ^S^ and σ^E^ transcription was dependent on ppGpp during both phosphate and amino acid starvation, DksA was required for σ^S^ under both conditions but dispensable for σ^E^ response to phosphate limitation [10].

The broad repertoire of DksA targets is due to its unusual properties. Although the presence of a zinc finger initially suggested that, similarly to most initiation factors, DksA may recognize specific DNA elements in its target promoters [1], the structure of *E*. *coli* DksA [13] revealed striking similarities to a family of regulators that control transcription by directly binding to bacterial RNA polymerase (RNAP) [18,19,20]. These regulators have a common two-domain organization; structurally similar coiled-coil (CC) domains extend through the secondary channel towards the active site of RNAP, whereas dissimilar globular domains bind outside the channel. Acidic residues located at the tip of the CC domains in *E*. *coli* DksA and Gre factors and *Thermus thermophilus* Gfh1 approach the active site, enabling their defined regulatory functions [13,19,21,22].

The regulatory specificity of the secondary channel factors is maintained, in part, by their preferential interactions with a subset of transcription complexes. For GreB, a conformational change in RNAP is thought to enable activity on paused, backtracked complexes [23]. Similarly, two reports [24,25] suggest DksA binds to various transcription complexes with different affinities, which could, in principle, direct DksA to specific targets in the cell. However, observations that cellular levels of DksA and Gre factors remain constant throughout cell growth [26] raise a question of whether their activity could also be modulated in response to cellular environment. For example, Gfh1 has been shown to flip between an active and an inactive conformation upon a pH shift [21]. Although neither the physiological role of Gfh1 nor the regulatory role of this transition is known, it could contribute to *T*. *thermophilus* adaptation to acidity; the authors speculated that analogous conformational switches may regulate activities of other secondary channel regulators. Consistent with this idea, *dksA* deletions in *Salmonella typhimurium* and *Shigella flexneri* compromise survival at low pH [8,27].

We report that DksA activity and binding to RNAP increase at lower pH. Our structural analysis suggests a pH-induced structural change in DksA that involves small modifications at the interface between the globular and the CC domains. Consistent with this hypothesis, changes at the interface abolish the characteristic pH-mediated regulation of DksA activity. We demonstrate that DksA is essential for *E*. *coli* survival under acidic conditions and that its cellular levels do not change under these conditions. Finally, we propose that DksA could serve as a pH sensor in the cell.

## Materials and Methods

### Reagents

All plasmids are listed in S1 Table. Oligonucleotides were obtained from Integrated DNA Technologies (Coralville, IA) or Sigma (St. Louis, MO), nucleotide triphosphates (NTPs) from GE Healthcare (Piscataway, NJ), ^32^P-NTPs from Perkin Elmer (Waltham, MA), restriction and modification enzymes from New England Biolabs (Ipswich, MA), PCR reagents from Roche (Indianapolis, IN), SYPRO Orange and other chemicals from Sigma. Plasmid DNAs and PCR products were purified using spin kits from Qiagen (Valencia, CA) and Promega (Madison, WI).

### Proteins

DksA variants and RNAP were purified as described previously [13,20]. HMK-tagged DksA was radiolabeled with [γ-^32^P]-ATP as described in [20]. Uniformly ^15^N-labeled and/or ^13^C/^15^N-labeled DksA (either WT or N88D) were purified from cells grown in M9 minimal medium supplemented with 1% (v/v) Eagle Basal Vitamin Mix (Life Technologies, Carlsbad, CA), containing 1g/L ^15^N ammonium chloride as the sole nitrogen source and, in ^13^C/^15^N samples, 3g/L ^13^C glucose as the sole carbon source. For WT DksA, we used a ^His10-TEV-^DksA construct (pRF2), which was obtained by cloning DksA WT from pVS11 into PstI and HindIII sites of pIA884, and for DksA mutant N88D, we used pIA1119, all of which are derivatives of pET28a (EMD Chemicals, Gibbstown, NJ). Protein production was induced with 0.2 mM IPTG for 7 h at 30°C. Cells were lysed by French Press in a disruption buffer (50 mM Tris-HCl pH 6.9, 150 mM NaCl, 0.1 mM EDTA, 1 mM 2-mercaptoethanol). Cleared lysate was loaded on a Ni-NTA agarose (GE Healthcare) column. After four consecutive washes with 10 vol of the disruption buffer containing 0, 50, and 100 mM imidazole (pH 7.5), DksA was eluted with 250 mM imidazole and loaded on a Resource Q column (GE Healthcare) equilibrated with HepA buffer (50 mM Tris-HCl pH 6.9, 0.1 mM EDTA, 1 mM 2-mercaptoethanol, 5% glycerol). Elution was carried out using 50–1500 mM NaCl gradient; DksA eluted around 350 mM NaCl. To remove the tag, purified DksA was incubated with the His-tagged TEV protease overnight at room temperature and loaded again on Resource Q column to separate the cleaved protein from the short His-tag, the TEV protease, and the uncleaved protein. Following TEV cleavage, the proteins have three additional residues (Gly-Leu-Gln) at the N-terminus.

### *In vitro* transcription assays

Transcription initiation assays were performed as described previously [28] in 20 mM Tris-HCl buffer pH 8.2 or 7.3 (measured at room temperature). IC_50_ was obtained by fitting the product band intensity *I* to the Langmuir binding equation: *I* = Baseline + (A*[DksA])/(IC_50_+[DksA]), where A is a proportionality constant, using the Scientist software (MicroMath).

### Binding assays

Localized Fe^2+^ cleavage assays were performed as described in [25] using ^32^P-labeled DksA^HMK^ and core RNAP. Titrations were performed using 1 nM DksA and 25, 50, 100, 200 and 400 nM RNAP in 10 mM Na-HEPES at different pH and 20 mM NaCl. Gels were dried and then visualized and quantified by phosphorimaging (ImageQuant). The fraction of cleaved DksA was plotted against RNAP concentration and fitted into a single binding site equation: K = (RNAP-X)*(DksA-X)/X, X = RNAP-DksA complex. Apparent Kd was calculated using the Scientist software for individual assays and averaged from at least three independent experiments.

### Circular dichroism (CD) and thermostability analyses

For CD analyses, DksA was dialyzed overnight into 20 mM phosphate buffers at pH 8, 7 and 6 with 50 mM NaCl. CD spectra were recorded using the Jasco J-815 Circular Dichroism Spectrometer from 200 μl samples. DksA concentrations were 50–100 μM. For thermostability analysis DksA concentration was reduced to 50 μM. CD was measured at 220 nm as a function of temperature (25–85°C); with 1°/min increments. The data were fit to the Gibbs-Helmholtz equation, assuming a ΔCp = 0, and linear baselines before and after the melting transition. That is, the temperature dependence of the CD of the folded and unfolded states was modelled as linearly dependent on temperature, with their own slopes and y-intercepts. The Kaleidagraph equation used was (m1+m5*(M0))+ ((m2+m6*(M0))-(m1+m5*M0))*1/(exp(m3*(1-(M0+273)/(m4+273))/1.987/(M0+273))+1); where m5 and m6 are the slopes of the folded and unfolded states, m1 and m2 their y-intercepts, m4 is the Tm and m3 is ΔH; M0 is temperature in degrees C. Absorbance at 600 nm was recorded simultaneously to monitor protein aggregation. No changes in A_600_ were observed for any of the variants. To further exclude this possibility, DksA renaturation following gradual temperature shift from 85 to 25°C was monitored. Consistent with the lack of aggregation, the CD spectra were nearly superimposable before denaturation and after renaturation.

### Differential scanning fluorimetry

The assay was performed as described in [29]. DksA (50 μM) was mixed with 5X SYPRO Orange (the stock concentration is not disclosed by the manufacturer) in 50 mM HEPES buffer at pH 6, 7 and 8 with 100 mM NaCl. The 25 μl reaction was incubated in a C1000 Thermal Cycler (Bio-Rad, Hercules, CA) for 25 minutes at 25°C followed by a gradual increase (1°C/30 sec) in temperature; with fluorescence intensity recorded every 1°. The unfolding temperature (Tu) is defined as the temperature at which the fluorescence increase reaches the half-maximum, and was estimated separately from three independent experiments and then averaged.

### NMR analysis

NMR studies as a function of pH were performed on uniformly ^15^N-labeled WT DksA in 20 mM sodium phosphate, 200 mM NaCl, 0.02% NaN_3_, 10% D_2_O at either pH 6 or 8 at 298K on a Bruker DRX-800 equipped with a 5 mm triple-resonance cryoprobe and z-axis gradient. For backbone assignments, we used the DksA^N88D^ variant, whose ^15^N-HSQC spectra are nearly identical to WT but provide higher resolution data (S1 Fig.). DksA^N88D^ NMR samples contained 0.4 mM protein, 20 mM sodium phosphate, 200 mM NaCl, 1 mM DTT, 50 μM ZnCl_2_, and 0.02% NaN_3_ at pH 6.0. The sequential backbone assignments were obtained from triple-resonance three-dimensional HNCO, HNCA, HNCACB and CBCA(CO)NH NMR spectra recorded on [U-^13^C/^15^N]-DksA-N88D at 298K on a Bruker DRX-600 (same probe as the DRX-800), and a 3D ^15^N-edited NOESY (200 ms mixing time) recorded on a ^15^N-labeled sample at 298K on the Bruker DRX-800. Data were processed with NMRPipe and analyzed with NMRViewJ. Chemical shifts were referenced directly (^1^H, ^13^C) or indirectly (^15^N) to the external standard 2,2-dimethyl-2-silapentane-5-sulfonate (DSS). Amide ^1^H and ^15^N chemical shift changes were quantified as: Δδ (ppm) = (Δδ _H_^2^ + Δδ _N_^2^/25)^1/2^ [30].

### Growth assays

Acid-survival was assayed as described in [27]. WT and Δ*dksA* cells (CH458 and CH2294, a gift from Christophe Herman) from an overnight culture grown in LB medium at pH 7.8 were diluted 1:50 into LB at pH 2.5. The medium was titrated to the desired pH using HCl or NaOH followed by filtration through a 0.2 μm filter. At different time points (0, 0.5, 1, 2, 4 and 8 h), aliquots were removed and serial dilutions were plated onto LB plates. Percentage of survival was determined using viable count after 16 h incubation at 37 ^o^C from three independent experiments. For pH adaptation experiments, cells from an overnight culture were diluted 1:50 into LB at pH 7.8, grown for 2 hours, diluted 1:50 into LB at pH 6.5, 5.5 or 4.5, grown for additional 2.5 hours, and diluted again 1:50 into LB at pH 3.5. Survival was determined as described above.

### Western blotting

WT (CH458) cells and Δ*dksA* (CH2294) cells were grown overnight in LB media, pH 7.8. The cultures were then diluted (1:25) into LB at pH 2.5 (at time 0) and grown at 37^0^C. 100 ml samples were collected at ½, 1, 2 and 4 hour time points. The cells were pelleted by centrifugation, washed three times with 500 μl of 50 mM Tris–HCl, pH 7.9, 100 mM NaCl, 5% glycerol, 0.1 mM DTT, 0.1 mM EDTA, protease inhibitors (Roche-complete ULTRA tablets, Mini EDTA-free) to remove traces of acidic media, resuspended in 50 μl of same buffer, and disrupted by sonication. The extracts were cleared by centrifugation, and total protein concentrations were determined by Bradford assay. Each lane was loaded with 5 μg protein. Proteins were separated on a 4–12% SDS Bis–Tris gel (Invitrogen, Carlsbad, CA, USA), transferred onto Trans-Blot nitrocellulose membrane (Bio-Rad Laboratories, Hercules, CA, USA) in Tris–glycine buffer, pH 8.3, containing 20% methanol at 100V for 1.5 h in a Mini Trans-blot Electrophoretic Transfer Cell (Bio-Rad laboratories). The blots were blocked overnight in 1× PBS-T, pH 7.5, 0.025% Tween 20 containing 5% nonfat dry milk. Following incubation with rabbit anti-DksA polyclonal antibodies diluted 1:500 in Phosphate-Buffered Saline-Tween (PBS-T) for 2 h at room temperature, the membrane was washed and probed with rabbit IgG (GE Healthcare) for 1 h (1:5000 dilution in PBS-T), washed again and exposed to ECL detection reagents (GE Healthcare). Imaging was carried out on Chemi-doc XRS^+^ Molecular imager (Bio-Rad).

## Results

### DksA is essential during acid stress

**Fig 1:**
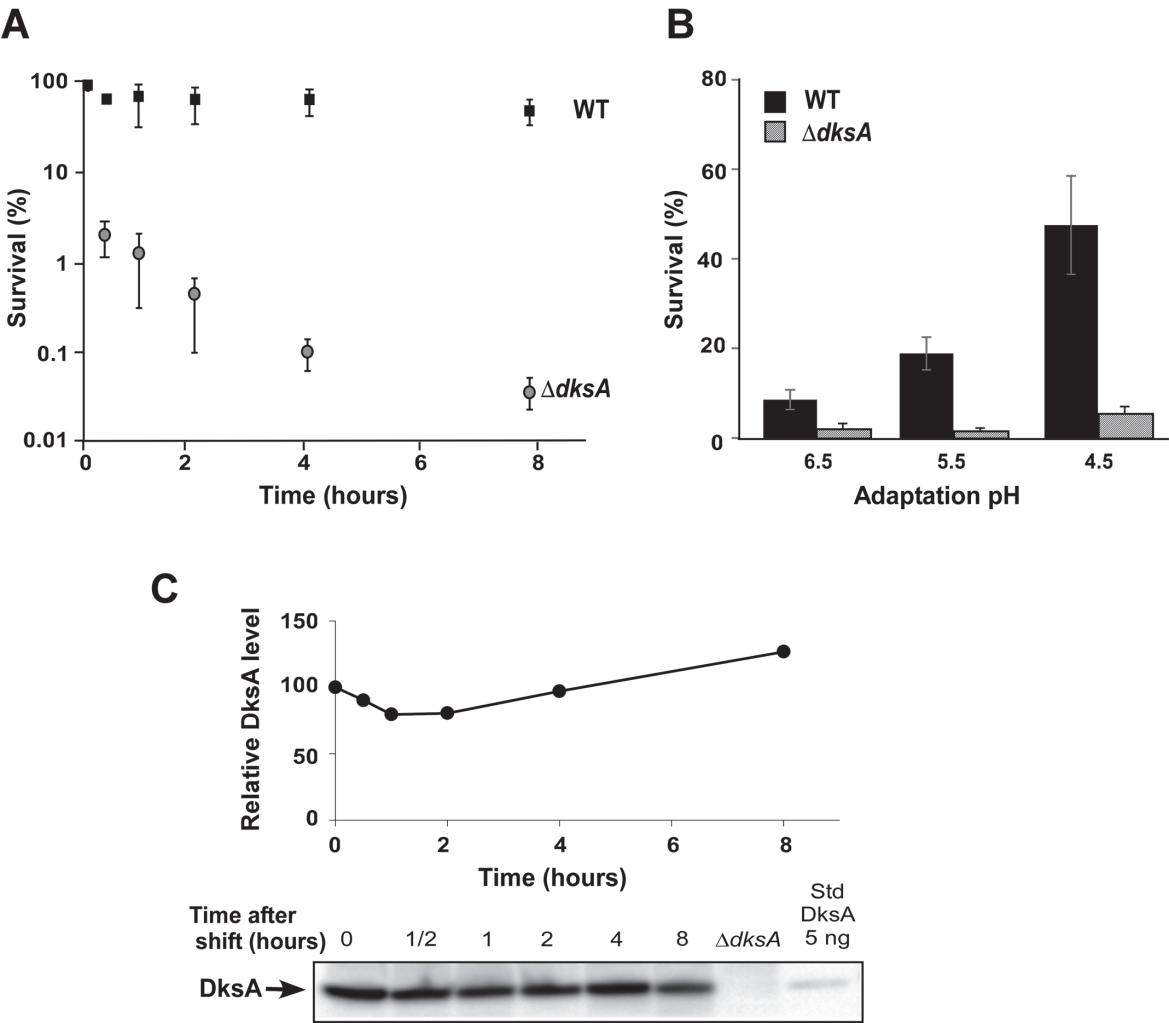
Δ*dksA* mutants are sensitive to acidic conditions. (A) WT and Δ*dksA E*. *coli* strains were grown overnight in rich medium at pH 7.8. Cultures were diluted 1:50 into LB medium at pH 2.5. At selected time points aliquots were taken and the percentage of survival of bacteria was determined using viable count. (B) WT and Δ*dksA E*. *coli* strains were grown at pH 7.8, followed by 2.5 hour adaptation at pH 6.5–4.5, and then diluted into LB medium at pH 3.5. Survival was determined using viable counts; the result after 2 hour incubation at pH 3.5 is shown. (C) DksA concentration remains relatively constant at low pH. Samples taken at different time points after a change in pH were analyzed using Western blotting with anti-DksA antibodies. Extract from the Δ*dksA* strain and purified DksA were loaded as controls.

A hypothesis that pH-dependent conformational switches in the secondary channel regulators may play a role in adaptation to pH stress, suggested by a conformational change observed in Gfh1 upon pH downshift [21], is consistent with earlier reports that *S*. *typhimurium* and S. flexneri ΔdksA mutants are sensitive to low pH [8,27]. However, growth *profiling [31]* did not reveal pH-dependent phenotypes for the Δ*dksA E*. *coli* strain. Therefore, we decided to directly test the role of DksA in response to acid stress in *E. coli*. We grew *E. coli* cells overnight at pH 7.8 and then diluted them into LB, pH 2.5. Next, we measured survival at different time points by plating aliquots at pH 7.8. Fig. 1A shows that, in contrast to the WT *E. coli* cells which persisted even after an 8-hour exposure to acid stress, the Δ*dksA* cells were extremely sensitive to the pH change, with 98% of cells dying within 30 minutes of exposure to low pH.

To test if DksA affects cell adaptation to acidic conditions, we grew cells at lower pH (6.5, 5.5 or 4.5) before diluting them to pH 3.5 (more surviving cells, as compared to pH 2.5, enable more reliable measurements). We observed increased survival during gradual pH adaptation in both the wild-type and Δ*dksA* cells (Fig. 1B). Thus, the requirement for DksA is not alleviated by adaptation.

This result might be explained by a rapid and dramatic change in DksA levels in cells exposed to low pH. However, the steady-state levels of DksA remained relatively unchanged even after 8 hours at lower pH (Fig. 1C). Another possibility is that pH-dependent structural changes enhance DksA activity at low pH.

### DksA activity is sensitive to pH

**Fig 2:**
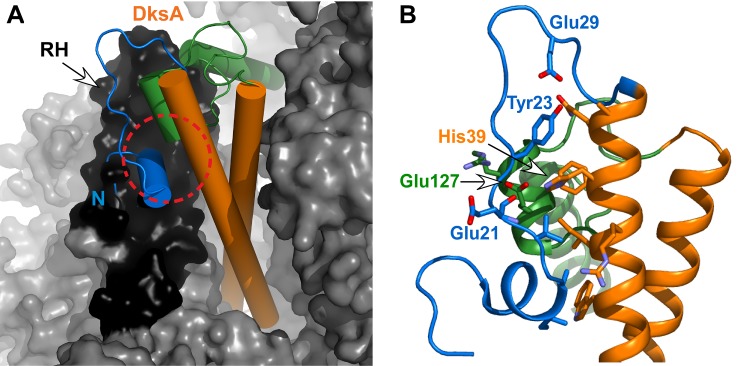
Conformational changes may be required to accommodate DksA in the secondary channel. (A) Hybrid model of DksA (PDB id: 1TJL_a) docked in the secondary channel of the *E*. *coli* RNAP core (PDB id: 3LU0). Clash between N-terminus and rim helix (RH) implies required conformational change and remodeling of domain interface (circle). (B) DksA structure reveals a large interface between the globular domain of the protein and parts of the CC domain. Residues that can contribute to the interdomain interactions are indicated. The CC domain is shown in orange, the N-terminal region in blue, and the C-terminal region in green.

Two recently proposed models generated by docking DksA into hybrid electron microscopy/X-ray models of *E*. *coli* RNAP suggest that the main binding site of DksA is a helix-hairpin-helix domain of the β' subunit (rim helices, RH) located at the entrance to the secondary channel [24,25]. Both models, though different in some respects, feature a clash between the N-terminal helix of DksA and the RH, suggesting that DksA might undergo conformational changes in order to productively bind to RNAP (Fig. 2A). Consistent with the models, a deletion of the N-terminal 18 amino acids of DksA increases the activity of the protein (26). The crystal structure of DksA reveals a large interface between the N-terminal and C-terminal regions and the CC (Fig. 2B) that contains charged or ionizable residues; one possible explanation is that changes in the protonation state of interface residues may mediate repositioning of the N-terminal helix in a manner that favors productive interaction with RNAP.

**Fig 3:**
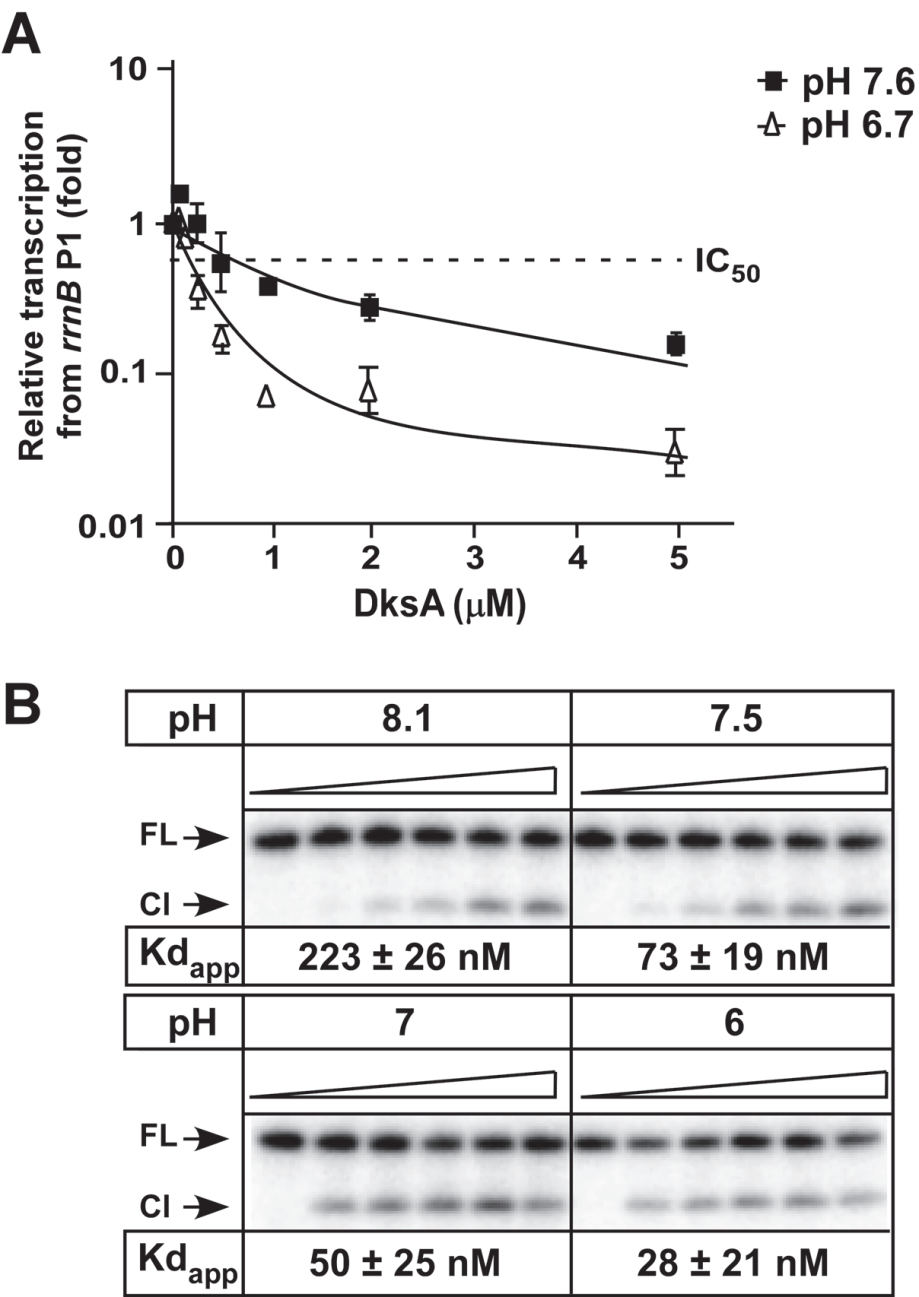
DksA is sensitive to changes in pH. (A) DksA activity increases at low pH. Increasing concentrations of DksA were added to holo RNAP (30 nM), ApC dinucleotide (0.2 mM), UTP (0.2 mM), GTP (4 μM) and [α-^32^P]-GTP (10 μCi of 3000 Ci mmol^−1^) followed by incubation for 15 minutes in Transcription buffer (20 mM Tris-HCl pH 7.9, 20 mM NaCl, 10 mM MgCl2, 14 mM 2-mercaptoethanol, 0.1 mM EDTA). A linear DNA fragment containing the *rrnB* P1 promoter was added to initiate transcription and the formation of a 4 nucleotide RNA product was monitored on a denaturing 8% acrylamide gel. A dotted line marks the inhibition of 50% of transcription and is denoted as IC_50_. The IC_50_ values (calculated using a single-site binding equation from three independent repeats combined in a best-fit curve, in μM) were: pH 7.6 − 0.7 ± 0.28, pH 6.7 − 0.11 ± 0.016. (B) DksA affinity to core increases at lower pH. DksA binding to core RNAP was performed using the localized Fe^2+^ mediated cleavage assay at different pH. DksA concentrations were: 0, 25, 50, 100, 200 and 400 nM. FL—Full length protein, Cl—cleaved protein, Kd app—apparent Kd.

To test whether DksA activity is pH dependent, we measured *in vitro* transcription from the *rrnB* P1 promoter, one of the main cellular targets for DksA [1]. Although DksA may affect several steps during transcription [1,13,32], its best characterized effect occurs during initiation [1]. To eliminate possible post-initiation effects of DksA, we measured the formation of a short transcript. We incubated increasing concentrations of DksA with RNAP, ApC, UTP and [α-^32^P]-GTP for 15 minutes prior to the addition of a linear *rrnB* P1 template, and monitored the formation of a 4 nucleotide-long RNA product. The relative transcription was plotted against DksA concentration to determine IC_50_ (Fig. 3A; see Materials and Methods for details). At pH 7.6, DksA inhibited transcription from the *rrnB* P1 with an IC_50_ of 0.7 μM. Reducing pH increased the inhibitory effect by DksA, lowering the IC_50_ to 0.11 μM at pH 6.7. Notably, RNAP activity in the absence of DksA did not change significantly in this range of pH values (S2 Fig.), suggesting that the IC_50_ changes are due to changes in DksA (or its interactions with RNAP) rather than a change in the transcription complex.

We next tested whether the effect of pH on DksA activity can be observed at other promoters. Lyzen et al. showed that DksA (when present at < 1μM) had a 3-fold stimulatory effect at the λP_R_ promoter at pH 8 *in vitro* [33]. We also observed a stimulatory effect of DksA at pH 7.6 at λP_R_ (S3 Fig.); however, this effect was reduced at higher DksA concentrations and was completely abolished at 5 μM. Interestingly, reducing pH reversed the effect of DksA at the λP_R_ promoter from stimulation to inhibition of transcription with an IC_50_ of 1.5 μM at pH 6.7.

### DksA binding to RNAP is sensitive to pH

Changes in the affinity of DksA for RNAP were shown to correlate with changes in its activity [34]. We measured the binding of DksA to core RNAP at different pH values using localized Fe^2+-^mediated cleavage (Fig. 3B). In this assay, hydroxyl radicals generated by Fe^2+^ bound in place of the catalytic Mg^2+^ ion in the RNAP active site induce cleavage of macromolecules within a ~10 Å radius. In a functionally bound to RNAP DksA which is labeled for visualization purposes, the CC tip is located close to the active site and is cleaved [13]. The fraction of cleaved protein is then plotted against increasing RNAP concentrations and fitted to the Langmuir binding equation, from which an apparent dissociation constant (Kd app) is derived [35]. The maximum cleavage of DksA was around 20% of the total protein and was constant at different pH. In addition, we measured binding affinities using a previously developed [25] fluorescence anisotropy assay (S4 Fig.) that measures functional as well as any non-functional binding of DksA; in the latter mode, DksA may be bound to the RNAP surface without entering the secondary channel. These different binding modes were reported previously for the yeast functional homolog of GreA, TFIIS [36]. Using both approaches, we found that the affinity of DksA for core RNAP increased dramatically upon shift from pH 8.1 to pH 6 (Fig. 3B and S4 Fig.).

### DksA stability is reduced at low pH

**Fig 4:**
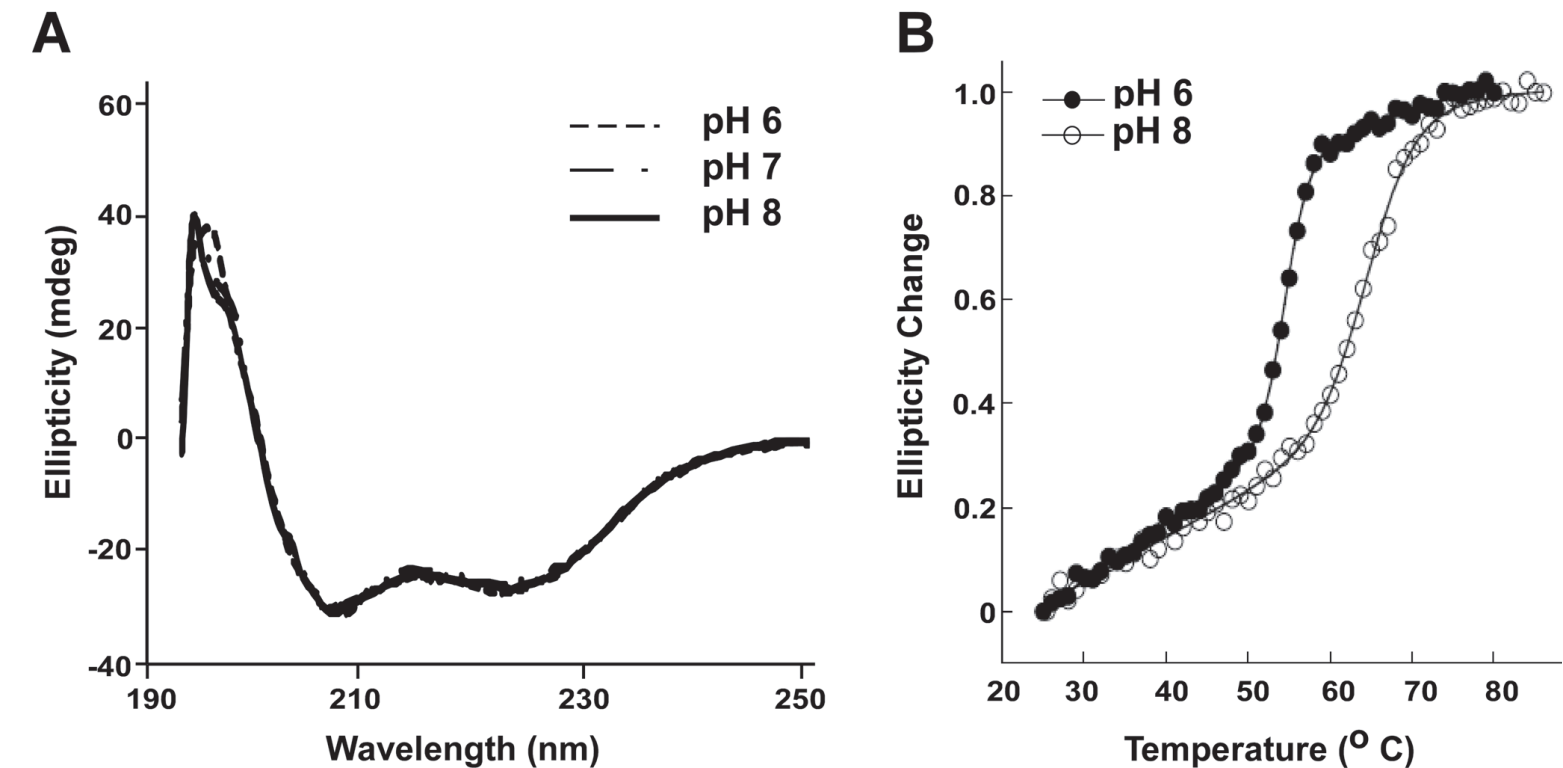
Effect of pH on DksA global structure and stability. (A) CD spectra recorded at room temperature using 50 μM DksA in phosphate buffer at different pHs. (B) Normalized ellipticity at 220 nm during a 1 degree/minute increase in temperature. Data were fit to the modified Gibbs-Helmholtz equation with linear temperature dependence before and after the transition region. Melting temperatures were 55 and 65°C at pH 6 and 8, respectively.

The increased affinity of DksA for RNAP could result from structural changes in either or both components. However, the analogy to Gfh1 motivated us to investigate whether DksA may exist in a more ‘active’ form at lower pH. To examine possible changes in its secondary structure, we recorded CD spectra of DksA at pH 8, 7 and 6 (Fig. 4A). As expected from the high proportion of helical structure, the CD spectra are dominated by these features (i.e., minima at 208 and 222 nm). We did not observe significant changes in DksA spectra as a function of pH, indicating that under those conditions pH does not significantly perturb the average secondary structure. Henard et al. have recently reported that DksA activity is regulated during oxidative and nitrosative stress using a thiol switch mechanism, wherein changes in the cysteine residues that comprise the zinc finger alter the zinc on/off rate and consequently DksA secondary structure and activity [9]. Since our data revealed no pH-dependent changes in the CD spectra, we conclude that the pH-induced change in DksA activity is not regulated by the same mechanism and does not involve cysteines modifications.

To investigate a potential pH-dependent change in the tertiary structure of DksA, we measured the protein stability using CD thermal melt assay and a differential scanning fluorimetry assay, which detects the increased fluorescence that accompanies binding of a hydrophobic dye SYPRO Orange to unfolded protein regions [29,37]. Both assays showed reduced DksA stability at pH 6 compared to pH 8 (Fig. 4 and S4 Fig.). In contrast, *E*. *coli* σ^70^ showed a very modest pH-induced shift in its unfolding temperature (S4 Fig.). The differential scanning fluorimetry and the CD melt show large differences in the melting temperature of DksA at both pH 8 and 6; 65 and 55°C as measured by CD vs 54 and 42°C measured by differential scanning fluorimetry. Such differences in melting temperatures are usually indicative of unfolding in two steps [38] and it is tempting to speculate that DksA unfolding involves changes in its tertiary structure followed by an alteration of its secondary structure.

### DksA exhibits structural changes at low pH

**Fig 5:**
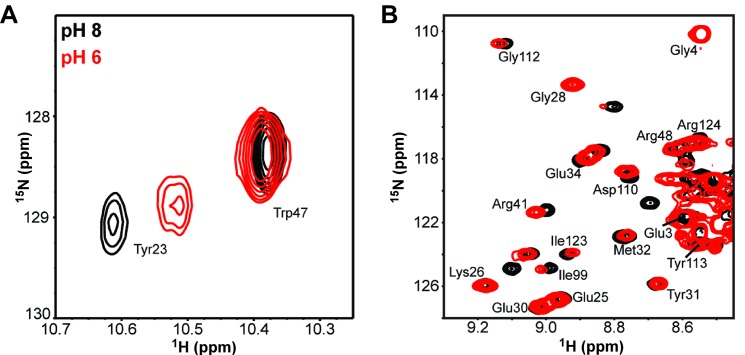
DksA structure is sensitive to pH. Two-dimensional ^1^H-^15^N HSQC spectra at pH 8 (black) and 6 (red) reveal large chemical shift changes at (A) Tyr23 and (B) many other residues.

To probe pH-induced structural changes in DksA, we collected two-dimensional ^1^H-^15^N correlated NMR spectra at different pH values (Fig. 5). Upon changing from pH 8 to pH 6, a number of amide signals are significantly shifted. Backbone resonance assignments were obtained through analysis of triple resonance NMR spectra recorded at pH 6; excessive line broadening and resonance overlap allowed only partial assignments (94 of 149 amides could be assigned). Significant perturbations were observed for the backbone amide signals of residues in the interface between the protein CC and the N- and C-terminal regions (e.g., Tyr23, Gln24, Asn33, Glu34, Gln36, Phe40, Arg41, Ile43, and Leu44; Fig. 5B and S5 Fig.). These localized chemical shift perturbations indicate localized conformational differences between pH 6 and 8.

### Histidine 39 plays a role in the pH sensitivity of DksA

**Fig 6:**
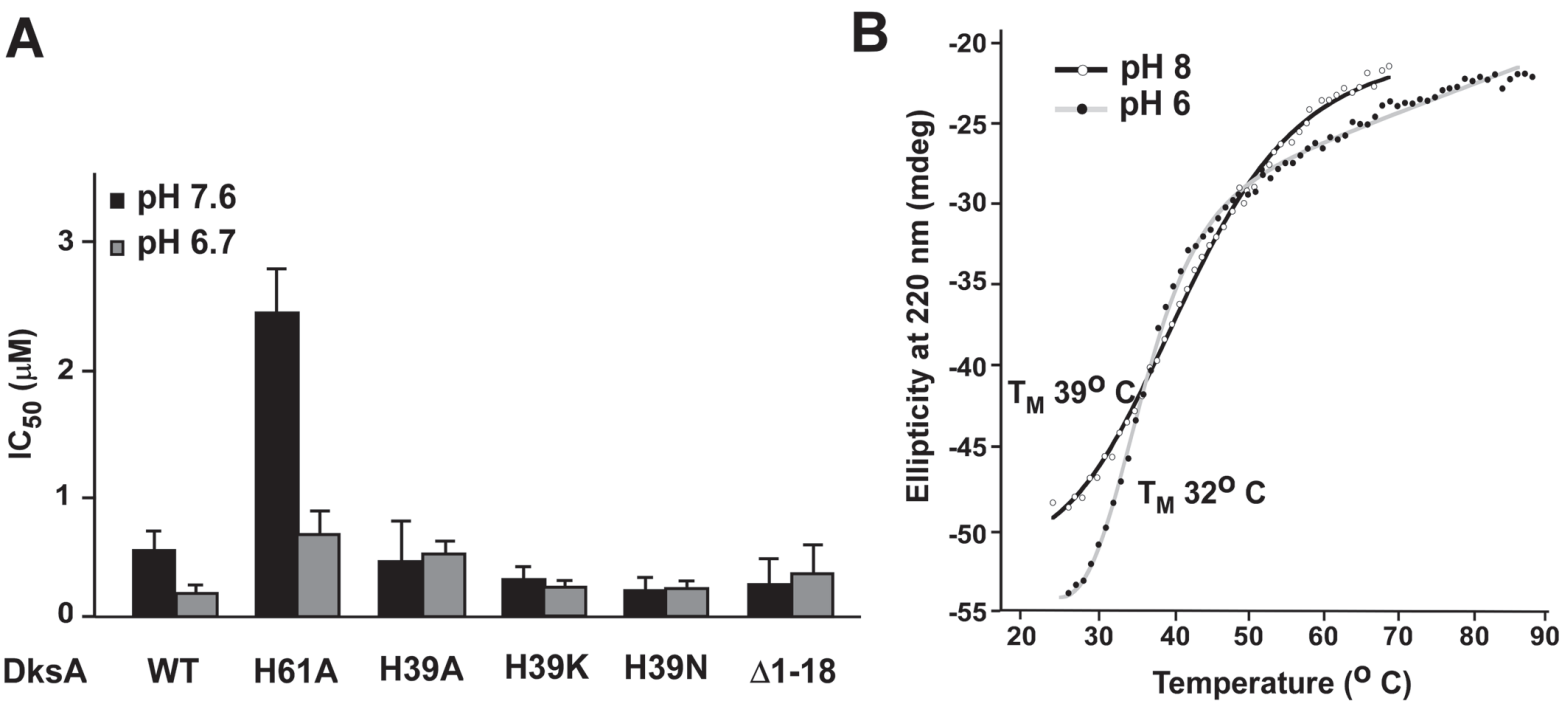
Substitution of His39 alters DksA sensitivity to pH. (A) The effect of pH on DksA variants. DksA activity and IC_50_ calculations were determined as described in Fig. 3A with the *rrnB* P1 promoter. Experiments were performed at least three times at each pH. (B) Thermostability of DksA^H39A^ is low and relatively insensitive to pH. Thermostability was determined as described in Fig. 4.

To probe the significance of pH-dependent changes at the interface between the C- and N-terminal regions revealed by NMR, we wanted to test whether changes at the interface would abrogate the pH- dependent change in DksA activity. Small C-terminal deletions completely abolish DksA activity [25] and hence cannot be tested. By contrast, a deletion of N- terminal 18 residues increases the protein activity at both *rrnB* P1 and λP_R_. Our results demonstrate that this deletion variant is not sensitive to pH (Fig. 6A and S6 Fig.), supporting the hypothesis that the N-terminal region is involved in the pH response of DksA.

The activity of DksA changes within pH range of 7.6 to 6.7, near the pKa values of free histidines. DksA contains two histidine residues, at positions 39 and 61. His61 is located in the middle of the CC domain and does not interact with other DksA regions whereas His39 is positioned at the interface between the two domains (Fig. 2B) and makes contacts to residues in both the N- and C-terminal regions. To determine whether these residues contribute to the pH-dependent changes in DksA, we substituted His39 and His61 individually with alanine. We first monitored transcription from *rrnB* P1 and λP_R_ promoters at different pH. The DksA^H61A^ variant exhibited a slightly reduced activity (relative to the WT protein) but displayed a similar dependence on pH, whereas the DksA^H39A^ variant was insensitive to pH changes at both promoters (Fig. 6A and S6 Fig.). Changing His39 to the polar amino acids, Asn and Lys, also abolished the pH sensitivity at *rrnB* P1 (Fig. 6A).

CD analysis of the H39 variants indicated that they share a similar secondary structure as the WT, albeit exhibit dramatic reduction in their stability (S6 Fig.). This result suggests that the substitution abrogates intramolecular interaction that helps stabilizing the tertiary but not the secondary structure of the protein.

A single Asn for Ile substitution at the 88 position of DksA increases the activity of the protein and its binding to RNAP [34]. We explored a hypothesis that the N88I substitution might lock DksA in an active conformation that can otherwise be achieved by reducing pH. We measured the activity of DksA^N88I^, its affinity for core RNAP, and its stability at different pH. We found that DksA^N88I^ responds to pH similarly to the WT protein (S7 Fig.), suggesting that the N88I substitution and pH affect DksA activity through different mechanisms.

## Discussion

DksA regulates a large set of genes during amino acid, iron, nitrogen, phosphate and carbon starvation, and is required for the proper regulation of gene expression by ppGpp, possibly the most universal stress regulator in bacteria [12]. Interestingly, while expression of some DksA-like proteins may be induced only during the time of need [28], the levels of DksA in *E*. *coli* are kept constant by a negative feedback mechanism during different growth phases [39]. Consistently, we did not observe dramatic changes in DksA levels upon pH downshift for at least 8 hours (Fig. 1C). Condition-specific regulation by DksA might be explained by at least two mechanisms. First, DksA function may be regulated at the level of activity through a conformational switch, as recently suggested by Henard et al. [9] and as was previously proposed for Gfh1 [21]. Second, the primary function of DksA could be to sensitize RNAP to changes in the levels of ppGpp and NTPs, whose fluctuations account for the rapid responses of DksA-regulated promoters to changes in nutritional conditions. Both mechanisms could be used to control diverse sets of DksA-dependent genes because coordinated activities of DksA and ppGpp are required for response to some stress cues but not others. Indeed, DksA has been shown to act independently of ppGpp at some promoters [4,6,33].

We show that DksA activity is stimulated by a pH downshift and that this change correlates with a stronger affinity for RNAP. We demonstrate that the structure and stability of DksA are sensitive to pH and propose that lower pH favors a more active DksA state. Observations that substitutions of His39 and the deletion of the N-terminal region eliminated DksA activation at low pH suggest that a change in the position of the N-terminal region may account for this effect. Changes in the protonation state of His39 will potentially affect hydrogen bonding between the imidazole side chain and the backbone carbonyl of Glu21 and the side chain carboxylate of Glu127, and between this same carboxylate and the backbone amide of Tyr23. This hypothesis is consistent with a steric clash hypothesized to hinder DksA binding to RNAP and is supported by a large pH-dependent chemical shift change in the ^15^N-HSQC spectra at Tyr23 located in the N-terminal region.

The His39 mutant is less stable yet as active as the WT protein at pH 8 but, unlike WT, fails to exhibit an increase in activity at pH 6. Both proteins show some reduced stability at lower pH, but in the case of H39A, the apparent Tu approaches the assay temperature, raising a question if the lack or H39A activation under acidic conditions could be due to its destabilization. Although this is possible, the pH-dependent change in apparent stability of the H39A variant is very small, and it is not clear how absolute Tu value measured with an isolated protein is related to the transcription activity. For example, efficient transcription initiation at the *lac*UV5 promoter is observed at temperatures that exceed Tu for σ^70^ measured by this assay [40].

Although our data argue that His39 is involved in the conformational change, other changes may also occur at lower pH. For example, zinc coordination may be jeopardized at lower pH, destabilizing the DksA Zn finger. However, several lines of evidence are inconsistent with this scenario: (i) the pKa of cysteines (8.4; [41]) is above the pH range tested; (ii) we did not observe chemical shift in C114 and in the areas surrounding the cysteines; (iii) contrary to what we would expect, lower pH increases the WT protein activity; and (iv) we did not detect pH-dependent changes in the CD spectra reported to be altered by changes in the cysteines [9].

At present, we cannot exclude a possibility that residues other than H39 contribute to pH-induced conformational changes that lead to changes in DksA activity and apparent stability. Additional experiments and higher resolution structural information, which at present seems limited by the intrinsic flexibility of the protein, will be required to fully develop the relevant mechanistic hypothesis.

### Acidic pH may mimic effects of ppGpp on DksA binding to RNAP

The mechanisms that govern the synergistic effects of ppGpp and DksA are presently unclear. One suggestion is that ppGpp and DksA allosterically increase each other’s affinity to RNAP, potentiating their respective effects on gene expression [34,42]. Consistent with this idea, two *dksA* alleles can suppress the requirement for ppGpp during growth on a minimal medium without amino acids [34]. The encoded changes were found to increase the binding of DksA to RNAP, suggesting that an increased affinity of DksA for RNAP is sufficient to suppress some of the effects of *relA*/*spoT* (ppGpp^0^) deletion in the cell. Likewise, overexpression of DksA can partially suppress the growth defects of ppGpp^0^ mutants [43]. Locations of the DksA- and ppGpp-binding sites on RNAP [14,24,44,45] exclude the possibility of their direct interaction and point towards a model in which one regulator favors binding of the other to RNAP allosterically. Our data suggest that a similar outcome (increase in DksA binding to RNAP) could be achieved by a conformational change in DksA which occurs during acid stress. Hence, DksA may serve as a direct sensor that is “turned on" by, and allows RNAP to respond to, changes in the cellular conditions rapidly.

### DksA role during acid stress

*E*. *coli* grows over a wide range of pH values and its own metabolism shifts the internal pH away from either extreme, depending on available nutrients and electron acceptors [46]. *E*. *coli* internal pH can shift significantly, from 4.7 to 7.8, when external pH changes from 2.5 to 6.9 [47]. The ability of some *E*. *coli* strains to survive exposure to strong acidic conditions is relevant for pathogenicity since bacteria have to overcome the acidic barrier of the stomach. Four overlapping systems are known to be involved in regulation of pH homeostasis in *E*. *coli*: a glucose-repressed system and three amino acid decarboxylase-dependent systems [46]. Each of these are regulated by σ^S^, an alternative σ factor required for gene expression during the stationary phase, thereby making the acid stress response a growth phase-dependent process [48].

Our data demonstrate that *E*. *coli* DksA is essential for survival under acidic conditions and suggest that DksA activity is stimulated by a conformational change upon reduction of the cellular pH, as observed *in vitro*. Our results raise several key questions that need to be addressed. First, is DksA effect direct and what genes are involved? DksA has many documented effects on the σS regulon and could potentiate expression of known pH homeostasis systems. DksA also elevates amino acids expression, of which some, such as arginine and glutamate, promote the resistance of *E*. *coli* to acid stress [47]. Second, what is the mechanism underlying the increased DksA affinity for RNAP? Third, does DksA act alone or in concert with ppGpp? DksA, but not ppGpp, is required for σ^70^ response to phosphate starvation [10] and ppGpp levels do not increase under mild (pH 5) extracellular acidic conditions [49]. It is possible that DksA also functions as a stand-alone stress regulator during (presumably σ^S^-dependent) adaptation to acid stress.

## Conclusions

We show that DksA is essential for *E*. *coli* survival in acidic conditions and that DksA activity and affinity for RNAP are increased at lower pH. NMR data reveal pH-dependent structural changes centered at the interface of the N and C-terminal regions of DksA and changes near this interface abolish pH-dependent changes in DksA activity *in vitro*. Our results suggest that conformational switches in response to pH and other cellular cues could be common among the secondary channel regulators.

## Supporting Information

S1 Table Plasmids. Details for the plasmids constructed in earlier studies can be found in the cited works [13,25,28,50–52].
(DOCX)

S1 Fig DksA mutant N88D facilitated backbone assignments via NMR. Overlayed two-dimensional ^1^H-^15^N HSQC spectra of DksA WT (black) and N88D (orange) reveal that while the two variants of the protein yield nearly identical spectra, N88D provides higher resolution data, especially in overlapped regions of the spectra, allowing backbone assignments to be determined.
(TIF)

S2 Fig The overall transcription yield remains relatively constant at different pH. Transcription was carried out for 15 minutes at 37°C in the absence of DksA at different pHs from the *rrnB* P1 promoter. Samples were separated by electrophoresis on 8% polyacrylamide, 7 M urea gels, and dried gels were visualized and quantified by phosphorimaging. The average signal at pH 7.6 was referred to as 1 and was used to normalize the overall signal at pH 6.7 for each experiment. The average fraction of transcription at pH 6.7 relative to pH 7.6 (normalized transcription) was calculated from 5 independent repeats.
(TIF)

S3 Fig DksA activity at the λP_R_ promoter. Increasing concentrations of DksA were added to holo RNAP (30 nM), ApU dinucleotide and [α-^32^P]-GTP followed by incubation for 15 minutes. A linear DNA fragment containing the λP_R_ promoter was added to initiate transcription and the formation of a 3 nucleotide RNA product was monitored on a denaturing 8% acrylamide gel. A dotted line marks the inhibition of 50% of transcription and is denoted as IC_50_. The IC_50_ values (calculated using a single-site binding equation from three independent repeats combined in a best-fit curve, in μM) were: pH 7.6 − >7.5, pH 6.7 − 1.5 ± 0.45.
(TIF)

S4 Fig DksA has an increased affinity to RNAP at lower pH. DksA^A35C^ (10 nM) was labeled using Atto 488 as described previously (18) and incubated with increasing concentrations of RNAP in HEPES buffer, pH 7.9 and pH 6.9, at 30°C for 10 minutes prior to anisotropy measurements. K_d*app*_ was calculated from three independent measurements. K_dapp_ values at different pHs were: pH 7.9 − 160 nM ± 28, pH 6.9 − 48 nM ± 10. (B) Representative scan of the fluorescence emission of SYPRO Orange binding to DksA as a function of temperature. (C) Unfolding temperature (Tu) of DksA at each pH. Tu is the temperature at which fluorescence emission is half-maximal, implying that 50% of DksA is unfolded.
(TIF)

S5 Fig Chemical shift perturbations in DksA between pH 6 and 8. Per residue weighted average amide ^1^H and ^15^N chemical shift perturbations were calculated as Δδ (ppm) = (Δδ _H_^2^ + Δδ _N_^2^/25)^1/2^ for all residues for which assignments are available. Red line indicates the mean value plus one standard deviation.
(TIF)

S6 Fig Properties of DksA variants. (A) Transcription inhibition by DksA variants at the λP_R_ promoter at different pH was measured as in S3 Fig. The IC_50_ values were calculated based on a single exponential fit from three independent repeats. (B) Overlaid CD spectra of DksA^WT^ and DksA^H39N^ recorded at room temperature using 100 μM DksA in phosphate buffer pH 8vshow no difference in their secondary structure. Similar spectra were observed for other H39 variants. (C) Comparison of thermostability of DksA^WT^ and DksA^H39^ variants measured at 220 nm as described for Fig. 4. ΔEllipticity values were used at the Y axis to better align the different spectra and denote the change in ellipticity at increasing temperatures for each variant. Ellipticity was recorded at 220 nm wavelength. Each sample contained 50 μM DksA in phosphate buffer pH 8.
(TIF)

S7 Fig The effect of pH on DksA^N88I^ activity and stability. (A) The IC_50_ values for DksA inhibition at the *rrnB* P1 promoter were calculated as described for Fig. 5. (B) DksA^N88I^ affinity to core RNAP was calculated using the localized Fe-mediated cleavage, as described for Fig. 3C. (C) Thermostability of the DksA^N88I^ variant was determined using the differential scanning fluorimetry, as described for S4 Fig.
(TIF)
