# Supplementary material for: pH Dependence of the Stress Regulator DksA
Source: PLoS One. 2015 Mar 23;10(3):e0120746. doi: 10.1371/journal.pone.0120746 (PMC4370453; doi:10.1371/journal.pone.0120746)
Supplement: S1 Table — Details for the plasmids constructed in earlier studies can be found in the cited works [13,25,28,50–52]. (DOCX) [file pone.0120746.s001.docx]

**Supplementary Table 1. Plasmids used in this work.**

| **Name** | **Description** | **Source/reference** |
| --- | --- | --- |
| pIA226 | λP_R_ promoter | [[1](#_ENREF_1)] |
| pIA536 | *rrnB* P1 promoter | [[2](#_ENREF_2)] |
| pIA579 | P_T7_ promoter–CBD–HMK–*dksA* | [[3](#_ENREF_3)] |
| pIA840 | P_T7_ promoter–CBD–*dksA*^[N88I]^ | [[4](#_ENREF_4)] |
| pIA884 | P_T7_ promoter–His_10_–TEV–*rfaH* | [[5](#_ENREF_5)] |
| pIA1047 | P_T7_ promoter–CBD–*dksA*^[A35C]^ | [[4](#_ENREF_4)] |
| pIA1095 | P_BAD_ promoter control | This work |
| pIA1096 | P_BAD_ promoter–*dksA* | This work |
| pIA1119 | P_T7_ promoter–His_6_–TEV–*dksA*^[N88D]^ | This work |
| pIA1146 | P_T7_ promoter–CBD–*dksA*^[H39A]^ | This work |
| pIA1150 | P_T7_ promoter–CBD–*dksA*^[H61A]^ | This work |
| pIA1152 | P_T7_ promoter–CBD–*dksA*^[Δ1-18]^ | [[4](#_ENREF_4)] |
| pIA1187 | P_T7_ promoter–CBD–HMK–*dksA*^[H39A]^ | This work |
| pIA1192 | P_BAD_ promoter–*dksA*^[H39A]^ | This work |
| pRF2 | P_T7_ promoter–His_6_–TEV–*dksA* | This work |
| pVS10 | P_T7_ promoter–*rpoA*–*rpoB*–*rpoC–*His_6_; *rpoZ* | [[6](#_ENREF_6)] |
| pVS11 | P_T7_ promoter–CBD–*dksA* | [[3](#_ENREF_3)] |

CBD = intein – chitin binding domain cassette from pTYB (NEB)

TEV = a recognition sequence for TEV protease

HMK = a recognition sequence for heart muscle kinase
