# Supplementary figures and images for: pH Dependence of the Stress Regulator DksA
Source: PLoS One. 2015 Mar 23;10(3):e0120746. doi: 10.1371/journal.pone.0120746 (PMC4370453; doi:10.1371/journal.pone.0120746)

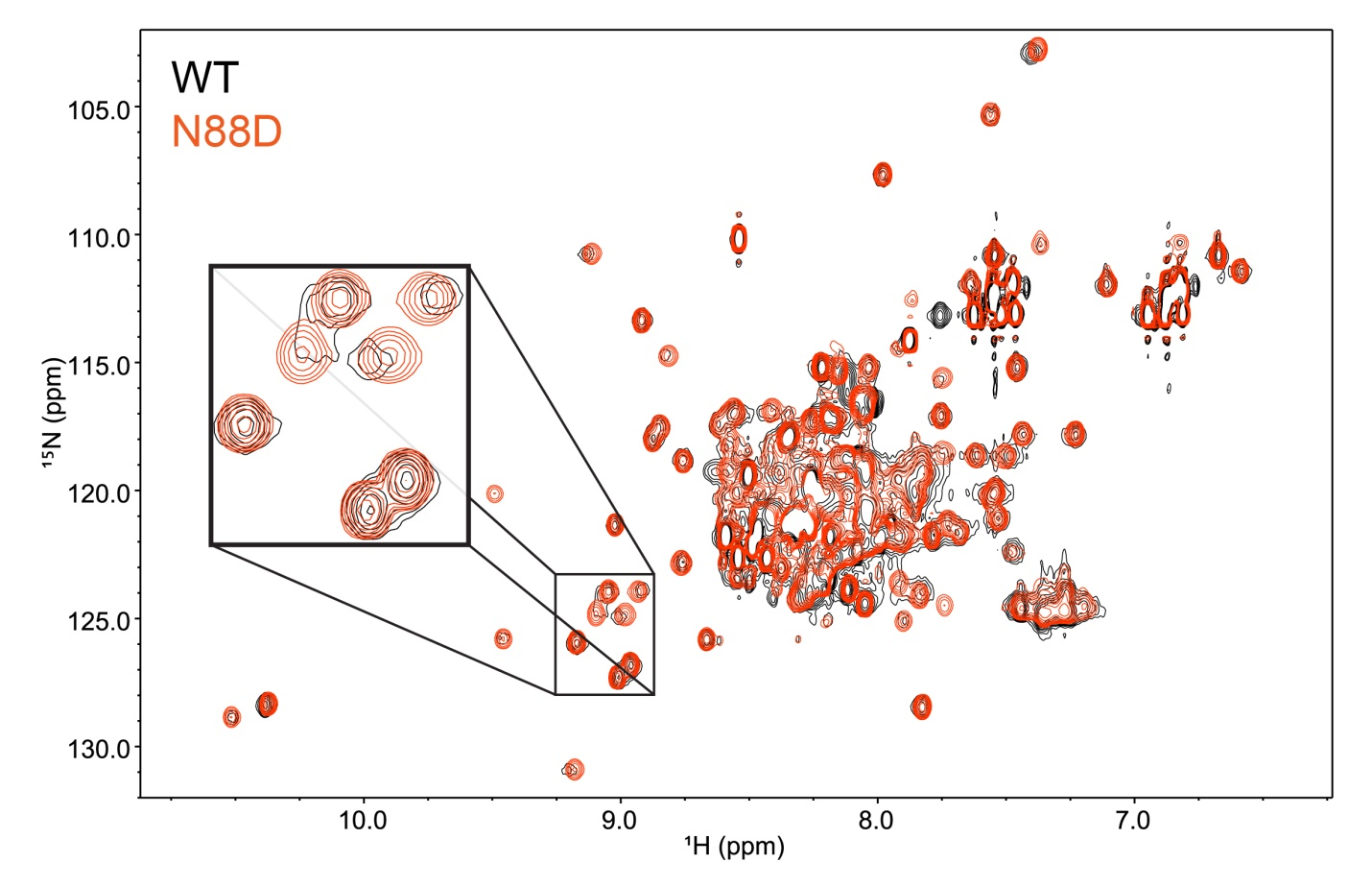

Supplement: S1 Fig — Overlayed two-dimensional 1H-15N HSQC spectra of DksA WT (black) and N88D (orange) reveal that while the two variants of the protein yield nearly identical spectra, N88D provides higher resolution data, especially in overlapped regions of the spectra, allowing backbone assignments to be determined. (TIF) [file pone.0120746.s002.tif]

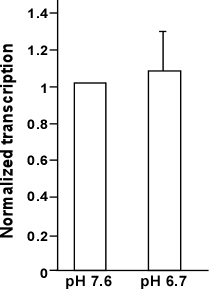

Supplement: S2 Fig — Transcription was carried out for 15 minutes at 37°C in the absence of DksA at different pHs from the rrnB P1 promoter. Samples were separated by electrophoresis on 8% polyacrylamide, 7 M urea gels, and dried gels were visualized and quantified by phosphorimaging. The average signal at pH 7.6 was referred to as 1 and was used to normalize the overall signal at pH 6.7 for each experiment. The average fraction of transcription at pH 6.7 relative to pH 7.6 (normalized transcription) was calculated from 5 independent repeats. (TIF) [file pone.0120746.s003.tif]

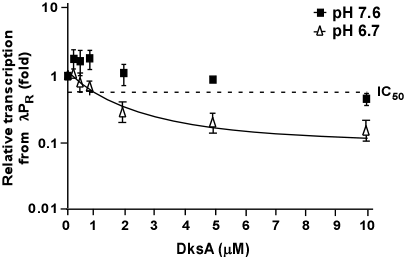

Supplement: S3 Fig — Increasing concentrations of DksA were added to holo RNAP (30 nM), ApU dinucleotide and [α-32P]-GTP followed by incubation for 15 minutes. A linear DNA fragment containing the λPR promoter was added to initiate transcription and the formation of a 3 nucleotide RNA product was monitored on a denaturing 8% acrylamide gel. A dotted line marks the inhibition of 50% of transcription and is denoted as IC50. The IC50 values (calculated using a single-site binding equation from three independent repeats combined in a best-fit curve, in μM) were: pH 7.6 − >7.5, pH 6.7 − 1.5 ± 0.45. (TIF) [file pone.0120746.s004.tif]

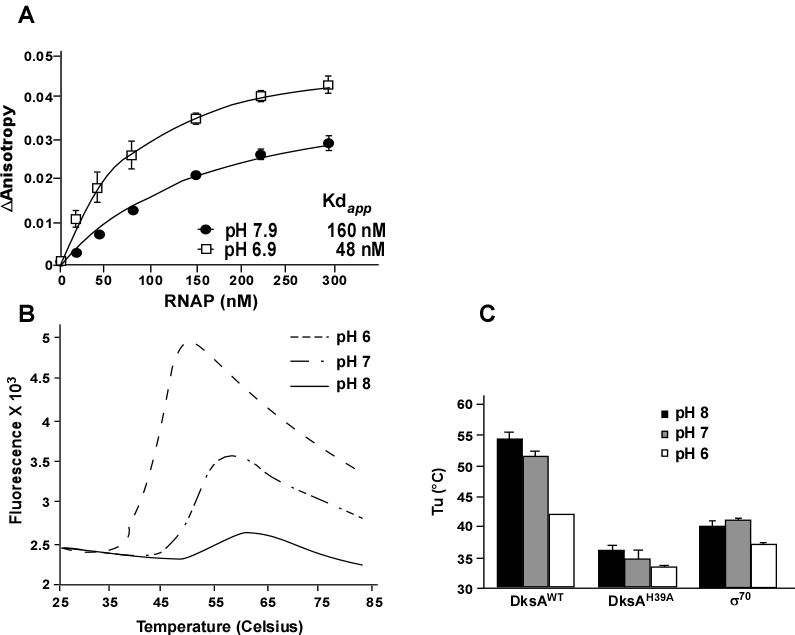

Supplement: S4 Fig — DksAA35C (10 nM) was labeled using Atto 488 as described previously (18) and incubated with increasing concentrations of RNAP in HEPES buffer, pH 7.9 and pH 6.9, at 30°C for 10 minutes prior to anisotropy measurements. Kdapp was calculated from three independent measurements. Kdapp values at different pHs were: pH 7.9 − 160 nM ± 28, pH 6.9 − 48 nM ± 10. (B) Representative scan of the fluorescence emission of SYPRO Orange binding to DksA as a function of temperature. (C) Unfolding temperature (Tu) of DksA at each pH. Tu is the temperature at which fluorescence emission is half-maximal, implying that 50% of DksA is unfolded. (TIF) [file pone.0120746.s005.tif]

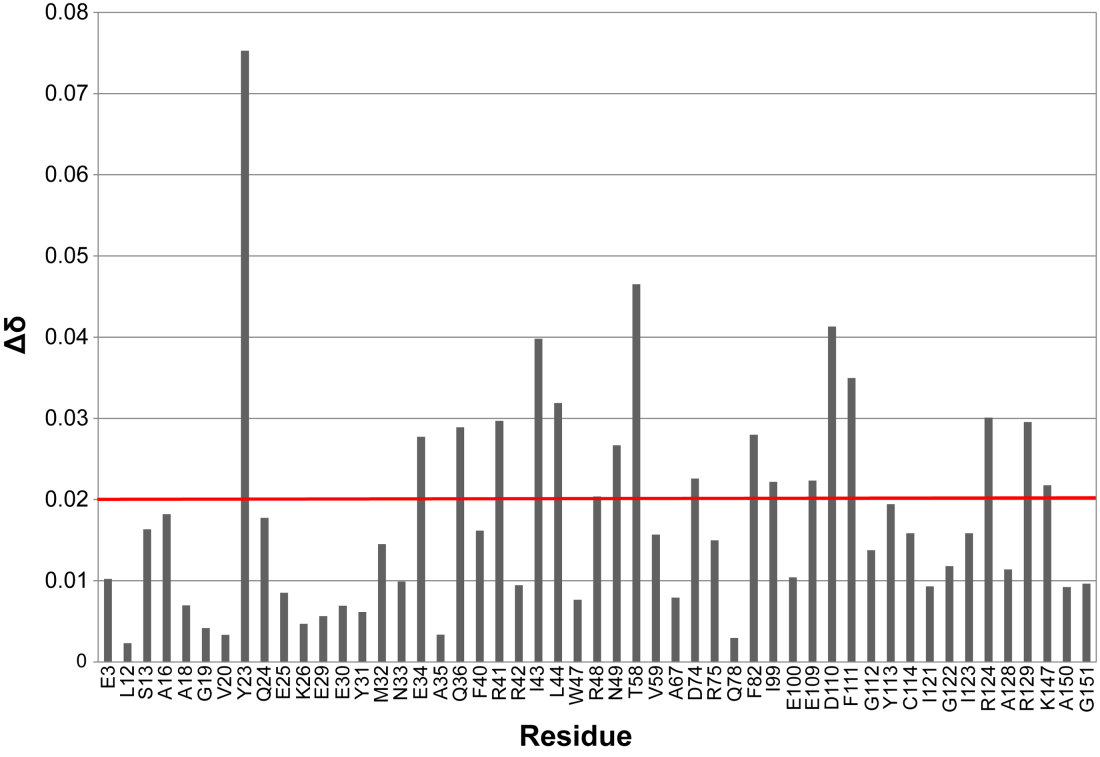

Supplement: S5 Fig — Per residue weighted average amide 1H and 15N chemical shift perturbations were calculated as Δδ (ppm) = (Δδ H 2 + Δδ N 2/25)1/2 for all residues for which assignments are available. Red line indicates the mean value plus one standard deviation. (TIF) [file pone.0120746.s006.tif]

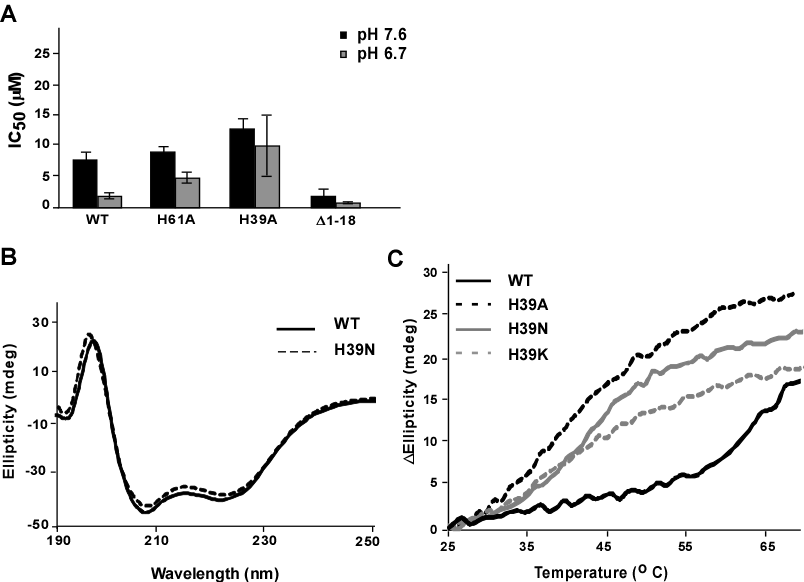

Supplement: S6 Fig — (A) Transcription inhibition by DksA variants at the λPR promoter at different pH was measured as in S3 Fig. The IC50 values were calculated based on a single exponential fit from three independent repeats. (B) Overlaid CD spectra of DksAWT and DksAH39N recorded at room temperature using 100 μM DksA in phosphate buffer pH 8vshow no difference in their secondary structure. Similar spectra were observed for other H39 variants. (C) Comparison of thermostability of DksAWT and DksAH39 variants measured at 220 nm as described for Fig. 4. ΔEllipticity values were used at the Y axis to better align the different spectra and denote the change in ellipticity at increasing temperatures for each variant. Ellipticity was recorded at 220 nm wavelength. Each sample contained 50 μM DksA in phosphate buffer pH 8. (TIF) [file pone.0120746.s007.tif]

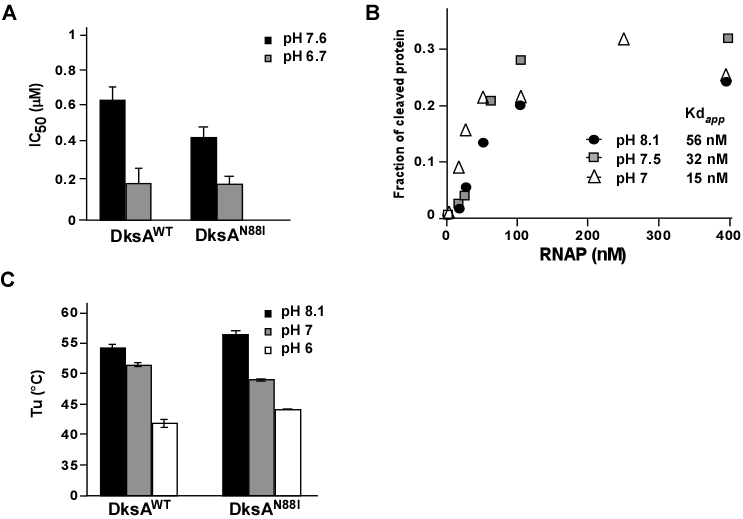

Supplement: S7 Fig — (A) The IC50 values for DksA inhibition at the rrnB P1 promoter were calculated as described for Fig. 5. (B) DksAN88I affinity to core RNAP was calculated using the localized Fe-mediated cleavage, as described for Fig. 3C. (C) Thermostability of the DksAN88I variant was determined using the differential scanning fluorimetry, as described for S4 Fig. (TIF) [file pone.0120746.s008.tif]
